# Task-Specificity of Muscular Responses During Motor Imagery: Peripheral Physiological Effects and the Legacy of Edmund Jacobson

**DOI:** 10.3389/fpsyg.2018.01869

**Published:** 2018-10-09

**Authors:** Jörn Munzert, Britta Krüger

**Affiliations:** Neuromotor Behavior Laboratory, Department of Psychology and Sport Science, Institute of Sport Science, Justus Liebig University Giessen, Giessen, Germany

**Keywords:** Jacobson, motor imagery, embodiment of cognition, electromyographic recording, psychophysiology

## Abstract

Motor imagery has become a key issue in cognitive neuroscience and particularly in fMRI research. However, peripheral physiological effects of motor imagery were already being studied a century ago with some research hypotheses even tracing back to Washburn (1916). This review focuses on research by Edmund Jacobson in the early 1930s. Jacobsen demonstrated that peripheral physiological effects rely on task-specific instructions: Bending the right arm elicits muscular responses in the right biceps, but not in the muscles of other limbs. This review discusses how Jacobsen examined this issue in a series of studies. This scientific spadework is worth recalling here because of its methodological innovations and its forward-looking discussion that even today, continues to be relevant for prospective research on this topic.

## Introduction

Edmund Jacobson was one of the first researchers to study the physiological effects of mental motor imagery in detail. Indeed, in this context, he is often cited as one of the basic references on the peripheral physiological effects of motor imagery. However, despite being cited frequently within the framework of motor simulation phenomena, the full extent of his research is rarely recognized. For example, few people know that he published not just one basic article on this topic (Jacobson, 1930a) but a body of six articles that systematically build on each other. This neglect of his work might be due to the strong recognition of his legacy in the field of relaxation techniques and specifically “progressive relaxation” (Jacobsen, 1929), also known as “progressive muscular relaxation.” This article presents a historical review of Jacobson’s early and experimentally farsighted work on motor imagery. The aim is to show how modern imagery research is built on the basic research he conducted in the 1920s and 1930s.

Edmund Jacobson (1888–1983) gained his Ph.D. in 1910 at Harvard University where he came into contact with William James. However, this contact seems to have had little influence on his own work. After receiving his Ph.D. at Harvard, he moved to Cornell University for 1 year where he joined Edward Titchener as a research fellow. Afterward, he taught physiology in Chicago until 1937. During this time, he also started to run his own laboratory in which he focused mainly on relaxation methods and their clinical applications^1^.

One of Jacobson’s central interests was in understanding how introspection might work. In principle, introspection procedures can be viewed as an intrinsic part of imagery research. However, the different research paradigms in imagery research indicate that interest does not always focus merely on introspective reports on the quality of imagery. Indeed, in most experimental settings, imagery procedures start normally with an explicit instruction to imagine a certain motor act (Munzert and Zentgraf, 2009). This implies at least some conscious effort (coupled to introspection) to produce and to control the respective image. However, this conscious effort can be reduced notably when imagery is instructed more implicitly, for instance, in mental rotation tasks for body parts (see Parsons, 1987). Participants in mental rotation experiments have to give a response after completing the mental rotation. Thus, even in this implicit method, there is a residual introspective component that is essential for the experimental procedure. The importance of introspection can be demonstrated more clearly for mental timing tasks in which the basic idea is to simulate a motor task internally and to signal the end of the action, for example in walking different distances (Decety et al., 1989; Munzert et al., 2015). Jacobson applied a similar paradigm within his experiments. He used a buzzer and instructed participants to imagine a specific movement upon hearing the first sound and to relax upon hearing the second sound (Jacobson, 1930a, p. 579). Hence, pairs of sounds signaled the start and end of the given motor imagery. To rule out the possibility that physiological effects were caused by the mere occurrence of the sound, on some control trials, he also instructed participants to relax between both signals.

As a physiologist, Jacobson was especially interested in measuring bodily reactions in response to mental activities. In his first 1930 paper, he argued that “if the study of mental phenomena is ever to become a true science, it would appear to be the task of the physiologist to bridge the gap to physics” (Jacobson, 1930a, p. 567). This led him to combine his genuine interest in mental activities, which were analyzed at least partly through introspective methods, with his interest in basic research in physiology and physics. Hence, Jacobson can also be viewed as an early promoter of embodied cognition phenomena.

## Fundamental Experiments on Motor Imagery

When appraising Jacobson’s experimental approach, one has to bear in mind that he was a true pioneer in the field of psychophysiology. Knowledge about psychophysiology in the early 1930s was far from the present understanding, and his attempt to bridge the gap between mental activities and peripheral physiological processes was ambitious, pioneering, and far ahead of its time. In his first 1930 paper, he followed a short theoretical introduction (referring to Weber, Fechner, and Wundt) by outlining the technical aspects of psychophysiological measurement on more than 11 pages. He elaborated the construction of his device extensively, referred to other measurement methods that were being introduced at that time, and discussed the meaning of the signals that were relevant in his experiments. Electrophysiology was a new and future-oriented approach, and Jacobson really pushed this approach in a new direction by, for example, discussing the properties of the string galvanometer (also known as the Einthoven galvanometer). In 1924, the Dutch physician Willem Einthoven (1860–1927) had received the Nobel Prize for Physiology and Medicine for developing this electrocardiological device. Metaphorically speaking, psychophysiological methods were appearing on the radar at that time.

The aim of the 1930 study was “to secure electrical records of neuromuscular states during specific acts of imagination” (Jacobson, 1930a, p. 569). Jacobson addressed this issue by using a sophisticated experimental device, as outlined above, and also by controlling task-related instructions. The basic experimental task contained the instruction to imagine bending the right arm continuously for some seconds. The start and the end were signaled by a buzzer. This condition was contrasted with several control conditions, for example, imagery of bending the left foot, the left arm, the relaxed or the paralyzed right arm; or extension of the right arm and general free imagery. Active movements of bending the left arm and the left foot were also included. When the specific conditions were assessed, 96% of the specific imagery trials (imagery of bending the right arm) were accompanied by significant electrical changes in the muscles of the right arm. In contrast, this effect was negative for 93% of the control conditions. When actual flexion was compared to imagined flexion for the right arm, actual flexion showed more electrical activity than the imagined contraction. This relation varied inter-individually, producing between 33 and 410% of stronger activation for active trials. The article presents averaged data for each single participant and also exemplary photographic records of electrophysiological measurements. Mere imagery of bending of the right arm elicited significant changes in the electrophysiological signal at the biceps area that was similar, but also weaker to that found in active trials for the same movement. This result demonstrated for the first time that the peripheral physiological effects of motor imagery are movement-specific and not a result of motor intentions in general.

In the following paper (Jacobson, 1930b), Jacobson reported electrophysiological data extending the range of tasks in which the right biceps was involved. He asked participants to imagine lifting a ten-pound weight with the right forearm. Control conditions referred to the instruction either not to imagine this task or to imagine the lifting movement with the other (left) arm. In 93% of experimental trials, he found a significant increase of muscular activation in the biceps, whereas no additional activation was found in control trials. Averaged for all participants, he found roughly a 450% increase of muscular activation in experimental trials compared to relaxation. This magnification ratio is higher than that in the bending task. This can be taken as supporting the argument that effort plays a significant role in motor imagery. Results are therefore in line with the work of Bakker et al. (1996) who showed that the imagined weight during a movement has an effect on EMG activity (see Mizuguchi et al., 2013 for a similar argumentation taking cerebral effects into account). A closer inspection of the time course of the electrophysiological data revealed an average time delay of about 400 ms, which can be interpreted as a reaction time (Jacobson, 1930b, p. 27). This might indicate that participants started to imagine the respective action shortly after the buzzer signal.

Additional data are reported in summary for very different imagery instructions such as writing one’s own name, boxing, or engaging in other actions using the right arm. The data demonstrated that the recordings from the biceps were stronger in imagining than in relaxation, but weaker than in bending the arm. There are also some references to recordings of alternating rhythmical movements such as climbing a rope, but the data are not reported in detail. These results underline once more the movement specificity of peripheral physiological effects relying on motor imagery.

In a subsequent series of control experiments, Jacobson tried to investigate whether the electrical signal recorded during imagery is a result of muscle contraction (Jacobson, 1930d). He presented data from four participants who were instructed to imagine bending the right or the left arm and lifting a ten-pound weight with the right or left arm. This time, he examined not only the electrophysiological effects in the biceps but also micro-movements of the right arm. The electrophysiological data underpinned the results of former studies, showing increased activation in the right biceps only for right-handed imagery. Furthermore, the movement recordings showed small microscopic flexions of the right arm that can be ascribed to contractions of the biceps muscle. Additional evidence is provided indicating that it is not possible to relax completely while simultaneously imagining a flexion. This conclusion differs from more recent studies that examined neural mechanisms of strength increases as a result of motor imagery and controlled for movement artifacts during imagery (Yue and Cole, 1992). More specifically, when examining mental strength training, it is mandatory to rule out any muscle contraction that could be connected to potential muscle hypertrophy (Reiser et al., 2011).

In a further study, Jacobson (1930c) reported electrophysiological recordings of eye muscles during visual imagery of large objects (the Eiffel Tower in Paris). He found that the recordings of the imagery data were similar to those for active movements. Jacobson reasoned that eye movements also occur during visual imagery (Jacobson, 1930c, p. 701). Reference values were again derived from relaxation phases; in this case, relaxation of the oculomotor system. As an early antecedent of oculomotor research in the context of motor imagery, results demonstrated task-specific eye movements during motor imagery with the eyes open and with closed eyes (Heremans et al., 2008). The results of his research on oculomotor effects led Jacobson to think about effects of visualizing motor acts such as bending the arm (Jacobson, 1931a). He invited three participants to take part in this study who “gave characteristically positive results in the form of action-potentials from the right biceps, when the instruction was merely, ‘Imagine bending the right arm”’ (Jacobson, 1931a, p. 119). Here it becomes clear that he used “imagination of bending the arm” in a way that focused on what we now define as motor imagery in contrast to visualization of a movement (see Munzert et al., 2009, for a discussion of motor imagery from a neuroscientific perspective). In the case of visualization of the movement, action potentials were absent from the biceps. On the other hand, instructions to visualize the motor action did elicit electrophysiological effects in the oculomotor region. Further control experiments showed that instructions for imagining bending the arm resulted in persistent activation of the biceps and movements of the eye in some but not all trials (Jacobson, 1931a, p. 120). It becomes unclear whether the latter effect was due to technical aspects of placing the needles close enough to the ocular muscle, or whether other systematic effects have to be considered. However, as a main result of this study, it can be seen that Jacobson (1931a) provided evidence for a fundamental difference between visual and motor imagery of movements.

We end our presentation of Jacobson’s imagery research with a single-case study. Here, Jacobson examined a patient who had suffered from a left arm amputation above the elbow (Jacobson, 1931b). Testing imagery for the right intact hand showed mixed results including 8 out of 12 trials showing a clear activation in the right biceps. A *post hoc* review revealed that this result could probably be ascribed to visualizing strategies especially in the first imagery trials (Jacobson, 1931b, p. 123). For imagery of bending the left affected hand, he reports results for electrodes placed at the left and the right arm. Imagery of bending the left arm showed significant action potentials in the left biceps on 13 out of 14 trials. Several control conditions elicited no or only minor electrophysiological activities in the left stump. This was different for imagining right-hand activities. Here, activities were also found for the left stump, indicating a more or less bilateral activation in both arms. A *post hoc* explanation that the participant mirrored right-hand mental activities to the left hand should be considered carefully. Bakker et al. (1996) also report some cross-talk of EMG activity to the contralateral limb while imagining lifting a weight. Research using functional magnet resonance imaging (fMRI) in amputees has shown a clear lateralized activation in motor areas for imagery and execution in both the intact and the phantom limb (Raffin et al., 2012). These and other recent studies on motor imagery with amputees can help to reinterpret Jacobson’s idea on mirroring mental movement activities. Whereas neural activation in cortical areas is clearly lateralized, there are also indications for a cross-talk to the opposite limb. It should be noted that Jacobsen was ahead of his time even in speculating about imagery of phantom limbs. He was the first to show that imagining a movement that cannot be performed due to amputation will still elicit the neural signals that would be necessary for its actual performance.

## Discussion

First, we have to point out that Jacobson did not aim to analyze motor imagery in a narrower sense. He was more interested in examining the peripheral physiological effects of differences between mental activities and relaxation states. Imagery of motor acts was the central task he used to specify the very broad term of mental activities. If today, we interpret his results as basic research on motor imagery, we should take this context carefully into account. His focus on the relaxation topic is underlined by the fact that he preferred to invite participants trained in relaxation techniques to perform his experiments. This does not influence the imagery strategies directly. In this perspective, it affects primarily the control condition of relaxation. The result is to increase the difference between experimental conditions (imagining of motor acts—general, mental activities) and control conditions (relaxation) by reducing muscular activity during relaxation. However, it has to be noted that studies investigating the influence of individual differences in imagery ability have found that participants with high imagery ability report significantly more relaxation than low imagers in relaxation tasks. Thus, imagery ability might be related to greater subjective responses to relaxation (Rickard et al., 1985; Johnsen and Lutgendorf, 2001). It can therefore be reasoned that Jacobson examined participants who might also have had the ability to create vivid mental images of the motor acts he was investigating.

A further interesting and experimentally farsighted manipulation was to implement different imagery modalities in his experiments. In contemporary literature, this is discussed as visual versus motor imagery (see Ruby and Decety, 2001, for a reference). In most of his studies, Jacobson implemented instructions that seem to aim toward imagery of the core motor act. However, in one study (Jacobson, 1931a), he focused on instructions that differed in terms of imagery modality. He compared motor-oriented instructions to instructions that were obviously focused on the visual imagery of a motor act. It is especially the “motor interpretation” of instructions that is clearly validated by electrophysiological data. In contrast, visual imagery of movements elicited activity in the oculomotor system, but showed no further activation in the target muscles that had proven to be activated during motor imagery. Furthermore, his data showed that it might sometimes have been difficult for his participants to clearly distinguish between motor and visual imagination.

However, the principal merit of his studies is that they revealed task-specific electrophysiological activation for imagery in those muscles that were active during actual execution of the specific motor task. He was not the first to formulate hypotheses on this issue (see Washburn, 1916). However, he developed methods with which to examine peripheral physiological effects. Modern research has underlined this effect of task-specific muscle activation during motor imagery (Bakker et al., 1996; Gandevia et al., 1997; Guillot et al., 2007, 2012). In particular, Guillot et al. (2007) have extended this issue by demonstrating that motor imagery is accompanied by subliminal EMG activity not only in the prime mover but also in related antagonists, synergists, and fixator muscles that work commonly as a muscle synergy. It has been further argued that this subliminal EMG activity results from central neural activation that is inhibited incompletely (Guillot et al., 2012).

Regarding recent neuroscientific research within the field, task specificity has become a key topic in research on motor imagery and action observation. In this context, newer approaches like multivoxel pattern analysis enable to identify task-specific neural patterns within one area of interest in fMRI studies (Gallivan et al., 2013; Filimon et al., 2014; Pilgramm et al., 2016). For example, it has been demonstrated that different actions as well as their modality (motor imagery, observation and execution) can be decoded from neural patterns in frontal and parietal motor cortices (Filimon et al., 2014; Zabicki et al., 2017). Again, this progress in neuroscientific research can be interpreted as a further underpinning of the forward-looking perspective that Jacobson offered so many years ago.

Jacobson interpreted the task-specific EMG activity as the outcome of a specific mental activity that he then could contrast with peripheral effects during relaxation. He was not concerned with the nature of motor imagery as we are nowadays when we refer to Jacobson’s pioneering work. Nevertheless, his experimental approach had great predictive power, because his results fit in nicely with such concepts as mental simulation theory (MST) based on the work of Jeannerod (1994). Jeannerod proposed that motor imagery is processed by an internal simulation of a motor act on the basis of motor representations in the motor-related cortical and subcortical brain areas (Munzert et al., 2009; Munzert and Zentgraf, 2009; Guillot et al., 2014; Eaves et al., 2016). Subliminal EMG activation, as demonstrated in Jacobson’s early experiments, is then interpreted as incomplete inhibition of the motor commands generated for simulation (Jeannerod, 1994; Bonnet et al., 1997; Guillot et al., 2007, 2012). MST offers a later neurophysiological explanation of the experimental results that Jacobson had collected in a series of experiments nearly 90 years ago. Jacobson’s great contribution was to acknowledge the central origin of the peripheral physiological signals collected during mental activities.

## Author Contributions

JM defined the aims of the research question and wrote the manuscript. BK contributed to all parts of the research question and to all parts of the manuscript.

## Conflict of Interest Statement

The authors declare that the research was conducted in the absence of any commercial or financial relationships that could be construed as a potential conflict of interest.

## Footnotes

1 Biographical data have been taken broadly from Gessel (1989).
